# Values and preferences of contraceptive methods: a mixed-methods study among sex workers from diverse settings

**DOI:** 10.1080/26410397.2021.1913787

**Published:** 2021-05-05

**Authors:** Euphemia Sibanda, Ania Shapiro, Bradley Mathers, Annette Verster, Rachel Baggaley, Mary E. Gaffield, Virginia Macdonald

**Affiliations:** a Project Director, The Centre for Sexual Health and HIV/AIDS Research, Harare, Zimbabwe (CeSHHAR Zimbabwe); Senior Lecturer, Liverpool School of Tropical Medicine, Liverpool, UK; b Consultant, World Health Organization, Geneva, Switzerland; c Technical Officer, World Health Organization, Geneva, Switzerland; d Unit Head, World Health Organization, Geneva, Switzerland; e Scientist, World Health Organization, Geneva, Switzerland

**Keywords:** sex workers, contraception, family planning, HIV, values and preferences, survey, focus group discussion

## Abstract

There is limited information on contraceptive values and preferences of sex workers. We conducted a mixed-method study to explore contraceptive values and preferences among sex workers. We conducted an online survey with individuals from 38 countries (*n* = 239), 6 focus group discussions (FGD, *n* = 68) in Zimbabwe, and 12 in-depth phone interviews (IDI) across 4 world regions, in June and July of 2019. Participants were asked about awareness of contraceptives, methods they had used in the past, and the determinants of their choices. Differences between respondents from high-, low- and middle- income countries were examined. Qualitative data were analysed thematically. Survey participants reported an awareness of modern contraceptive methods. FGDs found that younger women had lower awareness. Reports of condomless sex were common and modern contraceptive use was inconsistent. Determinants of contraceptive choices differed by setting according to results of the survey, FGD, and IDI. Regardless of country income level, determinants of contraceptive choices included ease of use, ease of access to a contraceptive method, and fewer side effects. Healthcare provider attitudes, availability of methods, and clinic schedules were important considerations. Most sex workers are aware of contraceptives, but barriers include male partners/clients, side effects, and health system factors such as access and clinic attitudes towards sex workers.

## Résumé

On dispose d’informations limitées sur l’avis et les préférences des professionnelles du sexe en matière de contraceptifs. Nous avons réalisé une étude à méthodologie mixte sur les préférences et la valeur que les professionnelles du sexe accordent aux méthodes contraceptives. Nous avons mené une enquête en ligne auprès de personnes originaires de 38 pays (*n* = 239), six discussions par groupe d’intérêt (*n* = 68) au Zimbabwe et 12 entretiens approfondis par téléphone dans quatre régions du monde, en juin et juillet 2019. Les participantes ont été interrogées sur leur connaissance des contraceptifs, les méthodes qu’elles avaient déjà utilisées et les déterminants de leurs choix. Les différences entre répondantes de pays à revenu élevé, intermédiaire et faible ont été examinées. Des données qualitatives ont été analysées par thème. Les participantes à l’enquête ont rapporté une connaissance des méthodes contraceptives modernes. Les discussions en groupe ont montré que les plus jeunes femmes étaient moins bien informées. Les rapports sexuels sans préservatifs étaient fréquents et l’emploi de contraceptifs modernes était irrégulier. Les déterminants des choix contraceptifs différaient d’après les contextes selon les résultats de l’enquête, les discussions de groupe et les entretiens approfondis par téléphone. Quel que soit le niveau de revenu national, les déterminants des choix contraceptifs comprenaient la facilité d’utilisation, la facilité d’accès à une méthode contraceptive et le moins d’effets secondaires. Les attitudes des prestataires des soins de santé, la disponibilité des méthodes et les horaires des établissements de santé étaient au nombre des considérations importantes. La plupart des professionnelles du sexe connaissent les contraceptifs, mais les obstacles incluent les partenaires/clients masculins, les effets secondaires et les facteurs relevant du système de santé comme l’accès et les attitudes des centres de santé à l’égard des professionnelles du sexe.

## Resumen

Existe información limitada sobre los valores y las preferencias de trabajadores sexuales con relación a anticonceptivos, por lo cual realizamos un estudio con métodos combinados para explorarlos. En junio y julio de 2019, realizamos una encuesta en línea con personas provenientes de 38 países (*n* = 239), seis discusiones en grupos focales (DGF, *n* = 68) en Zimbabue y doce entrevistas a profundidad (EAP) telefónicas en cuatro regiones del mundo. A las personas participantes se les preguntó si tenían conocimiento de los anticonceptivos, qué métodos habían usado en el pasado y cuáles eran los determinantes de sus elecciones. Se examinaron las diferencias entre informantes provenientes de países de altos, bajos o medianos ingresos. Se analizaron los datos cualitativos temáticamente. Las personas encuestadas informaron tener conocimiento de los métodos anticonceptivos modernos. Las DGF encontraron que las mujeres jóvenes tenían menos conocimiento. Informes de sexo sin condones eran comunes y el uso de anticonceptivos modernos no era sistemático. Los determinantes de las elecciones anticonceptivas diferían por entorno, según los resultados de la encuesta, DGF y EAP. Independientemente del nivel de ingresos del país, los determinantes de las elecciones anticonceptivas eran: facilidad de uso, facilidad de acceso a un método anticonceptivo y menos efectos secundarios. Las actitudes de los prestadores de servicios, la disponibilidad de métodos y los horarios de las clínicas eran consideraciones importantes. La mayoría de los trabajadores sexuales tiene conocimiento de los anticonceptivos, pero enfrenta barreras tales como parejas/clientes masculinos, efectos secundarios y factores del sistema de salud, entre ellos acceso y actitudes en la clínica hacia trabajadores sexuales.

## Background

Sex workers are disproportionately affected by HIV and sexually transmitted infections (STI).^1^ They also have a lower uptake of health services, including HIV testing and sexual and reproductive health (SRH) services, compared to the general population.^2^ Where sex work is criminalised, violence against sex workers is often not reported, not monitored, and hidden.^3^ Stigma, including self-stigmatisation, and discrimination among sex workers is common and complex, with many intersecting factors including racism, sexism, homophobia, poverty, and gender discrimination.^4^ Many sex workers report unwillingness to access health services due to fear of poor treatment by healthcare workers.^5–7^ The direct impact of criminalisation and repressive policing of sex work on both HIV and STIs has been shown.^8^ Community-led programmes for sex workers can help improve uptake of services, but in many settings these programmes lack funding.^9^

Uptake of modern contraception among women – including sterilisation, intrauterine devices (IUD), hormonal implants and injectables, oral contraceptive pills, male and female condoms, vaginal barrier methods (including the diaphragm, cervical cap, and spermicidal foam, jelly, cream, and sponge), the lactational amenorrhoea method, and emergency contraception – varies within and across geographic regions.^10^ In 2015, 28.5% of African women aged 15–49 reported the use of a modern contraceptive method, compared to 61.8% in Asia and 66% in Latin America and the Caribbean. Unmet need for contraception (the proportion of women who want to stop or delay childbearing but are not using any modern contraceptive method) is high among women of reproductive age in low- and middle-income countries. Estimates show that in 2019, 22 million women in Eastern Africa, 20 million in Western Africa, 23 million in South Eastern Asia, 28 million in Eastern Africa, and 87 million in Southern Asia had unmet contraceptive needs.^11^ Across regions, there is evidence that sex workers have greater unmet need for contraception than the general population, with estimates as high as 64% in Kenyan sex workers, 30% in Madagascar, 35% among adolescent sex workers in China^6–8,12–14^ and reports of over-reliance on condom-only use instead of the recommended dual protection.^9–15^

Rates of unplanned pregnancy and unsafe abortion among sex workers are high: 61% of sex workers had an unplanned pregnancy and 47% had had an abortion in a Zambian study;^16^ a systematic review found unintended rates of pregnancy among sex workers ranging from 7.2 to 59.6 per 100 person years;^17^ a Cameroonian study found that among more than 2000 sex workers, 57.6% reported history of unintended pregnancy and 40.0% reported prior abortion.^18^ There are similar findings in other countries.^19–21^ The situation is partly driven by poor access to health services and criminalisation or restriction of abortions in some settings. Unplanned pregnancies may also add financial pressures and lead to increased risk-taking. While modelling suggests an increase in condomless sex among sex workers on pre-exposure prophylaxis (PrEP), and potentially increased rates of unintended pregnancy, the scale-up of PrEP among sex workers presents opportunities for integrating contraceptive with PrEP and HIV services.^22^ Interventions which link contraceptive services and HIV services, including PrEP, show improvements in the use of modern contraceptives among sex workers.^23^ For contraceptive service attendees, those that perceive themselves at higher risk of HIV are more likely to access PrEP, but this group does not always include sex workers, who often report high rates of condom use with clients.^24,25^ Sex workers must be able to exercise their rights to determine the number and spacing of their children.

There is limited information on sex workers’ values and preferences related to contraception and how to optimise programmes to promote access to safe and effective contraceptive choices. Following the *Evidence for Contraceptive Options and HIV Outcomes* trial (ECHO), which showed no safety concerns with Depo-Provera (a long-acting contraceptive injection) among three long-acting reversible contraceptives (LARCs),^26^ WHO updated its medical eligibility criteria (MEC) for contraceptive use guidance. The MEC, the first edition of which was published in 1996, presents current WHO guidance on the safety of various contraceptive methods for use in the context of specific health conditions and characteristics. Understanding the values and preferences of end-users is an important part of this process. We sought to understand preferences among geographically diverse sex workers and to compare those from low- and middle-income countries, where sex work is more often criminalised and there is poorer access and lower contraceptive prevalence among women in general, and high-income countries. We conducted a study from June to July 2019 that explored contraceptive values and preferences among sex workers at high HIV risk.

## Methods

A mixed-methods study was conducted, including a multi-country survey, focus group discussions (FGDs) in Zimbabwe, and in-depth interviews (IDIs) in four geographical regions of the world. We defined sex workers as per the United Nations definition to include adults who receive money or goods in exchange for sexual services, either regularly or occasionally. We limited participation to those aged between 18 and 49, i.e. adults of reproductive age. For the FGD we only included cisgender female sex workers. For the online survey and in-depth interviews, we excluded cisgender male sex workers, but did not put any other limitations on gender. We only included participants who had engaged in sex work in the last 12 months.

### Online survey

An online survey was developed by the WHO consultant and WHO staff in headquarters and regional offices. SurveyMonkey questionnaires were administered in English, French, Russian, and Spanish. The survey was advertised in diverse networks including global, regional, and national sex worker networks (e.g. The Global Network of Sex Work Projects, Asia Pacific Network of Sex Workers, African Sex Workers Alliance, and others); UN agencies (WHO, UNAIDS, UNFPA); and other organisations (e.g. Frontline AIDS, LINKAGES, Centre for Sexual Health and HIV AIDS Research, LVCT Health, John Snow International, Re-Action South Africa, Reproductive Health Uganda, Médecins du Monde Myanmar, and others). The survey was advertised on relevant Twitter feeds, emailed directly to organisations, open to all sex workers of reproductive age capable of conceiving, excluding cisgender men, and anonymously completed. The survey was open from 17 June to 17 July 2019. Questions addressed knowledge and sources of contraceptives and factors promoting/limiting use, with over half of the questions presented in the form of Likert scales. There were 23 questions, including an indication of consent to participate in the study and it took around 20 min to complete (Supplement 1). Participants did not receive any reimbursement.

Data from the online survey were analysed using IBM SPSS Statistics version 26. Because provision and access to contraception may be affected by country income status, we examined differences between respondents from high-income countries (HIC) and low- and middle-income countries (LMIC) using Chi-squared tests of association for categorical variables and *t*-tests for differences in means for continuous variables.

### Focus group discussions

FGDs were held in June 2019 at “Sisters with a Voice” (the Sisters programme), the Zimbabwe national sex worker programme that is implemented by the Centre for Sexual Health and HIV AIDS Research (CeSHHAR) Zimbabwe on behalf of the Ministry of Health and Child Care.^27^ Free services provided to sex workers include education on safer sex with provision of condoms, STI treatment, contraception, HIV testing and counselling, PrEP, and referral and support for linkage to HIV treatment. CeSHHAR was chosen as a study partner for various reasons: Sisters with a Voice provides services to the majority of sex workers in Zimbabwe and has considerable geographic reach, being one of the only nationally scaled programmes for sex workers in Africa allowing for the participation of sex workers from a range of ages and background. Further, CeSHHAR has much experience conducting sex worker-led, qualitative research.

Purposive selection ensured inclusion of the following groups of women recruited from the Sisters programme: 25 years and older (two groups, *n* = 24), 20–24 years (one group, *n* = 10), and 16–19 years (two groups, *n* = 22). One group of women (various ages) who were not engaged with the Sisters programme (*n* = 12) was also recruited. Outreach workers and peer educators in the Sisters programme assisted with recruitment for both women attending the Sisters Programme and those not. It was important to include women not attending in order to capture a diversity of views given the expectation that perceptions could be shaped by sources/access to information and contraception.

Experienced researchers facilitated FGDs in Shona, the participants’ language. Each group was split into three sub-groups to encourage participation by all; role play elicited understanding of contraceptive decision-making among participants, followed by guided discussions of the role play and other themes including contraceptive practices, preferences, and experiences; role of clients/partners in contraceptive choices; and whether views differed by age and level of engagement in the Sisters programme. Each FGD lasted 90 min to two hours.

### In-depth interviews

IDIs were conducted with sex workers from four WHO regions (Europe – 3 participants; Africa – 4 participants; Western Pacific – 2 participants; and Americas – 3 participants) recruited through referrals from sex worker-led organisations and communities as described above for the online survey. While participants from other WHO regions, particularly the South East Asia and Eastern Mediterranean Regions, were sought, researchers did not identify any sex workers who wanted to participate during the study period, limiting participants to 12 individuals. Interview participants resided in Australia, Brazil, El Salvador, France, Kenya, Malawi, Russia, South Korea, Spain, Tanzania, the United States of America, and Zimbabwe, although, due to concerns about confidentiality, country of residence was not linked to interview responses. Twelve key informants were interviewed: 10 through Skype or telephone in English and Russian. Interpreters could not be sourced for Spanish and Portuguese, therefore respondents from Brazil and El Salvador answered the interview questions in written form and sent them back over email. All interviews used a standard interview guide containing follow-up probes exploring similar themes to the FGD and online survey. All interviews were audio-recorded and transcribed verbatim. Following transcription, qualitative data were inputted into a computer-assisted qualitative data analysis software, coded by theme, and thematically analysed using content analysis techniques. Each interview lasted between 20 minutes and one hour.

### Qualitative data handling and analysis

IDIs and FGDs were audio-recorded, transcribed, and translated into English as necessary. Analysis was conducted in parallel with data collection;^28^ field notes reflected emerging themes, and analytic summaries drew comparisons within and across groups. Thematic analysis principles guided the process:^20^ a coding framework was drawn from the summaries and discussions among the research team, and coding was done in NVIVO11^29^ (qualitative data handling and management software).

### Ethical considerations

Ethical approval was sought from the Medical Research Council of Zimbabwe, reference MRCZ/A/2474 (FGDs) and WHO Ethical Review Committee (survey and IDIs), reference ERC.0003195. Written informed consent was obtained from FGD participants before study procedures began. Survey and IDI participants were provided with an information sheet and consent form prior to beginning research activities, followed by electronic or verbal consent, respectively. As per standard procedure, participants were only given an opportunity to decide on participation when they demonstrated understanding of the study and what participation entailed. Questionnaires and qualitative data files were identified using only study identity numbers, rather than names, and were stored in password-protected computers with access restricted to study staff.

## Results

### Participants

The online survey was completed by 239 participants from 38 countries in all 6 WHO regions (Africa, Western Pacific, South East Asia, Eastern Mediterranean, Europe, and Americas). Of these, 107 (45%) were from LMIC, Table 1. Three participants who did not report country of residence were excluded from comparative analysis of HIC and LMIC.

**Table 1.** Online survey respondents characteristics

Characteristic	High-income countries *n* (%) [*n* = 129]	Low- and middle-income countries *n* (%) [*n* = 107]	Total *n* (%) [*n* = 239]^a^	*p*-value
**Average age(years)**	32.1	33.0	32.6	0.372
18–25	27 (20.9%)	25 (23.4%)	52 (21.8%)	0.495
26–30	36 (27.9%)	20 (18.7%)	57 (23.8%)	
31–35	26 (20.2%)	19 (17.8%)	46 (19.2%)	
36–40	22 (17.1%)	18 (16.8%)	41 (17.2%)	
>40	18 (14.0%)	25 (23.4%)	43 (18.0%)	
**Average number of children**	0.3	1.2	0.7	<0.001
0	104 (80.6%)	39 (36.8%)	143 (60.6%)	<0.001
1	12 (9.3%)	33 (31.1%)	45 (19.1%)	
2	13 (10.1%)	34 (32.1%)	48 (20.3%)	
**Education** None or primary school only High school Advanced education University of higher	0 (0) 36 (27.9) 28 (21.7) 65 (50.4)	13 (12.2) 32 (29.9) 28 (26.2) 34 (31.8)	24 (9.1) 77 (29.2) 58 (22.0) 105 (39.8)	<0.001

^a^ Includes three respondents not reporting country of residence and omitted from analysis of country income categories.

The average age of participants in the online survey was 32.6 years (range 19–49) with no significant difference between HIC and LMIC. All HIC participants and 88% from LMIC had at least high school education, *p* < 0.001. On average respondents from LMIC had more children than those in HIC (*p* < 0.0001; Table 1).

Among the 68 FGD participants, 55 (81%) were single, and the average age was 27 (range 16–54). The median number of children they had was 1 (range 1–5). Half of IDI participants were from LMIC. IDI participants were not asked to disclose information regarding their personal characteristics.

### Information on and awareness of contraceptive methods

Survey respondents most commonly accessed information through healthcare providers (67.8%, *n* = 162) and the internet (63.2%, *n* = 151). Only 2.5% (*n* = 6) of respondents reported that they do not access information on contraception. Those living in HIC more frequently reported accessing information from healthcare providers than participants in LMIC (76.0% vs 57.0%; *p* = 0.002). Those living in HIC also more frequently reported accessing information via the internet (72.1% vs 52.3%; *p* = 0.002). Respondents from LMIC more frequently reported accessing information through work (34.6% vs 17.1%; *p* = 0.002) or through outreach workers (23.4% vs 10.1%; *p* = 0.006) (Table 2).

**Table 2.** Information on, knowledge about, access to and use of contraceptive methods, results of online survey

		Low- and middle-income countries (*n *= 107)	High-income countries (*n* = 129)	High- vs low- and middle-income countries *χ*^2^*p*-value	Total (*n* = 239)^a^
**Where do you access information on contraception?** (Multiple responses possible)	At work	37 (34.6%)	22 (17.1%)	0.002	59 (24.7%)
	Drop-in centre	12 (11.2%)	40 (31.0%)	<0.001	52 (21.8%)
	Friends or family	33 (30.8%)	58 (45.0%)	0.027	91 (38.1%)
	Healthcare providers	61 (57.0%)	98 (76.0%)	0.002	162 (67.8%)
	Mobile clinic	17 (15.9%)	6 (4.7%)	0.004	23 (9.6%)
	NGO or CBO	44 (41.1%)	43 (33.3%)	0.217	87 (36.4%)
	Outreach worker	25 (23.4%)	13 (10.1%)	0.006	38 (15.9%)
	Internet	56 (52.3%)	93 (72.1%)	0.002	151 (63.2%)
	Don’t access	2 (1.9%)	4 (3.1%)	0.692	6 (2.5%)
**Where do you access contraceptives?** (Multiple responses possible)	At or through work	19 (17.8%)	19 (14.7%)	0.52	39 (16.3%)
	Pharmacy	67 (62.6%)	70 (54.3%)	0.195	138 (57.7%)
	Drop-in centre	9 (8.4%)	35 (27.1%)	0.000	45 (18.8%)
	Family planning clinic	28 (26.2%)	39 (30.2%)	0.491	68 (28.5%)
	Friends or family	6 (5.6%)	10 (7.8%)	0.798	17 (7.1%)
	Mobile clinics	19 (17.8%)	7 (5.4%)	0.003	26 (10.9%)
	NGO/CBO	34 (31.8%)	33 (25.6%)	0.015	68 (28.5%)
	Outreach workers	16 (15.0%)	11 (8.5%)	0.123	27 (11.3%)
	Private clinic or hospital	23 (21.5%)	21 (16.3%)	0.306	45 (18.8%)
	Public clinic or hospital	26 (24.3%)	48 (37.2%)	0.033	75 (31.4%)
	Other	4 (3.7%)	33 (25.6%)	–	38 (15.9%)
**Which contraceptive methods have you heard of?** (Multiple responses possible)	Male condoms	105 (98.1%)	127 (98.4%)	1.000	235 (98.3%)
	Female condoms	92 (86.0%)	122 (94.6%)	0.024	216 (90.4%)
	Diaphragm	27 (25.2%)	113 (87.6%)	<0.001	142 (59.4%)
	Non-hormonal (copper) IUDs	73 (68.2%)	121 (93.8%)	<0.001	196 (82.0%)
	Tubal ligation	78 (72.9%)	123 (95.3%)	<0.001	203 (84.9%)
	Vasectomy	67 (62.6%)	124 (96.1%)	<0.001	193 (80.8%)
	Traditional methods	63 (58.9%)	118 (91.5%)	<0.001	183 (76.6%)
	Oral pill	94 (87.9%)	125 (96.9%)	0.007	221 (92.5%)
	Contraceptive rings	38 (35.5%)	108 (83.7%)	<0.001	146 (61.1%)
	Hormonal contraceptive patches	34 (31.8%)	98 (76.0%)	<0.001	133 (55.6%)
	Hormonal IUDs	53 (49.5%)	111 (86.0%)	<0.001	165 (69.0%)
	Injectables	54 (50.5%)	101 (78.3%)	<0.001	157 (65.7%)
	Hormonal implants	52 (48.6%)	111 (86.0%)	<0.001	165 (69.0%)
	Emergency contraceptive	80 (74.8%)	124 (96.1%)	<0.001	206 (86.2%)
**Which contraceptives have you used in the last year?** (Multiple responses possible)	Male condoms	100 (93.5%)	122 (94.6%)	0.718	225 (94.1%)
	Female condoms	22 (20.6%)	34 (26.4%)	0.297	56 (23.4%)
	Diaphragm	1 (0.9%)	4 (3.1%)	0.381	5 (2.1%)
	Non-hormonal (copper) IUDs	6 (5.6%)	15 (11.6%)	0.106	21 (8.8%)
	Tubal ligation	5 (4.7%)	9 (7.0%)	0.456	14 (5.9%)
	Vasectomy	1 (0.9%)	10 (7.8%)	0.014	11 (4.6%)
	Traditional methods	20 (18.7%)	23 (17.8%)	0.864	43 (18.0%)
	Oral pill	29 (27.1%)	26 (20.2%)	0.209	55 (23.0%)
	Contraceptive rings	0 (0.0%)	5 (3.9%)	0.065	5 (2.1%)
	Hormonal contraceptive patches	1 (0.9%)	1 (0.8%)	1.000	2 (0.8%)
	Hormonal IUDs	3 (2.8%)	20 (15.5%)	<0.001	23 (9.6%)
	Injectables	13 (12.1%)	6 (4.7%)	0.035	19 (7.9%)
	Hormonal implants	7 (6.5%)	10 (7.8%)	0.720	17 (7.1%)
	Emergency contraceptive	22 (20.6%)	27 (20.9%)	0.944	50 (20.9%)
**How often do you use condoms with clients?**	All the time	87 (81.3%)	113 (87.6%)	0.031	203 (84.9%)
	More than half the time	10 (9.3%)	12 (9.3%)		22 (9.2%)
	Half the time	5 (4.7%)	1 (0.8%)		6 (2.5%)
	Less than half the time	2 (1.9%)	0 (0.0%)		2 (0.8%)
	Never	0 (0.0%)	0 (0.0%)		0 (0.0%)
	N/A	0 (0.0%)	3 (2.3%)		3 (1.3%)
	No response	3 (2.8%)	0 (0.0%)		3 (1.3%)
**How regularly do you use condoms with other male partners?**	All the time	34 (31.8%)	28 (21.7%)	0.03	63 (26.4%)
	More than half the time	25 (23.4%)	30 (23.3%)		56 (23.4%)
	Half the time	6 (5.6%)	14 (10.9%)		20 (8.4%)
	Less than half the time	14 (13.1%)	20 (15.5%)		34 (14.2%)
	Never	13 (12.1%)	24 (18.6%)		37 (15.5%)
	N/A	5 (4.7%)	13 (10.1%)		19 (7.9%)
	No response	10 (9.3%)	0 (0.0%)		10 (4.2%)

^a^ Includes three respondents not reporting country of residence and omitted from analysis of country income categories.

Respondents reported variable access to information on contraception. Multiple IDI participants noted inadequate access to accurate and reliable information, exacerbated by undertrained health staff, funding shortages, and/or restrictive national reproductive health policies.

“*Nowadays there is no information provided, because the president has limited and discouraged contraceptive methods* * … * *Any health information* * … * *has to be distributed by the Ministry of Health. Otherwise, it isn’t allowed for any other institution or organization to develop its own materials [regarding] health issues.*” (IDI participant, WHO African region)

Survey participants generally showed good awareness of available contraceptives. Respondents from HIC were more aware of different contraceptives than those from LMIC, most notably hormonal contraceptive patches (76.0% vs 31.8%; *p* < 0.0001), contraceptive rings (83.7% vs 35.5%; *p* > 0.0001), and diaphragms (87.6% vs 25.2%; *p* < 0.0001), Table 2.

However, pockets of misinformation exist. FGDs revealed lower knowledge levels among younger sex workers who found it difficult to ask for contraceptive advice:

“*Some are not able to ask [questions about contraception] because they may have started sex work at the ages of 12 years or 15 years. If you just approach an ordinary person [to ask for information] they will say, ‘Aah you are sexually active at your tender age!’. So they will be afraid.”* (19-year-old woman, FGD)

In addition, those not attending the Sisters programme were also misinformed about contraceptives.

“*Bicarbonate of soda is very effective [as a contraceptive]. As soon as I finish [having sex], before I eat anything I should [mix] a teaspoonful of bicarbonate of soda with [some] water, then drink. Immediately after* * … * *it will burst like boo-o-o, and the sperms will come out* … ” (54-year-old woman, not attending Sisters, FGD)

Of survey participants, the most frequently reported sources of contraceptives were pharmacies (57.7%; *n* = 138), public clinics or hospitals (31.4%, *n* = 75), and family planning clinics (28.5%, *n* = 68). Respondents from LMIC were more likely to report reported accessing condoms at non-government or community-based organisations (31.8% vs 25.6%; *p* = 0.015) and mobile clinics (17.8% vs 5.4%; *p* = 0.003) than those from HIC (Table 2).

### Contraceptive methods and regularity of use

Survey respondents reported using a variety of contraceptive methods, most commonly: male condoms (94.1%, *n* = 225), female condoms (23.4%, *n* = 56), oral contraceptives (23.0%, *n* = 55), and emergency contraception (20.9%, *n* = 50). Uptake of all other methods was low. There was little difference in the type of contraceptive used in the last year between HIC and LMIC, with hormonal IUDs more frequently reported in HIC (15.5% vs 2.8%; *p* = 0.001) and injectables more frequently reported in LMIC (12.1% vs 4.7%; *p* = 0.04) (Table 2). FGD participants most frequently reported use of the hormonal implant (39.7%; *n* = 27) and oral contraceptives (21.1%; *n* = 15). Twelve FGD participants did not use contraceptives (17.7%).

Survey respondents reported using condoms more regularly with clients than with other sexual partners. While 84.9% (*n* = 203) reported always using condoms with clients, only 26.4% (*n* = 63) reported always using them with other partners (Table 2). Results were comparable among HIC and LMIC respondents. The extent to which respondents use condoms in their personal lives may also depend on partner type. Generally, IDI participants preferred to use non-barrier contraceptive methods with long-term partners to reduce discomfort and/or enhance sexual pleasure. One key informant commented that condom use at work may influence contraceptive practices at home.

“*In my personal life, [with] a long-term partner, generally we don’t use condoms, if it’s someone that I’m seeing exclusively or monogamously. With short-term partners* * … * *it’s probably 85% of the time. Sometimes you just get caught up in the heat of the moment. And I think also because I use condoms so much at work, it’s almost a fetish for me, having unprotected sex, because it’s very forbidden* * … *” (IDI participant, Western Pacific)

Among FGDs respondents, condomless sex with clients was common, mainly because of financial pressure – clients either offered more money for condomless sex or walked away at the suggestion of a condom.

“*If he doesn’t want to wear the condom and you force him* * … * *as soon as you put the condom on him he loses the erection. Then he will say look, it’s not working so give me back my money* … ” (24-year-old woman, FGD)

Sometimes men forced or tricked sex workers into having unprotected sex:

“*He will make you bend over, saying ‘bend over I am putting the condom on’ … * *but he will then insert without* * … * *then suddenly you feel the wetness as he comes inside you and finishes.*” (22-year-old woman, FGD)

FGDs revealed that sex workers did not stop working in the absence of contraceptive protection; financial vulnerability forces them to risk the consequences. Some women reported occasionally using the withdrawal method, although many of them appreciated that this was unreliable.

“*I don’t let a client go just because I don’t have contraception/protection. I will pay special attention [during sex] and when he is about to come I will quickly get off so that it [semen] falls to the ground.*” (25-year-old woman, FGD)

FGD participants reported that unplanned pregnancies were common, with many accessing abortions. Given that abortion is restricted in Zimbabwe, women reported that abortions were unsafe and informal providers employed traditional methods including use of plant materials such as *the roots of a pepper tree*, *tea leaves*, and *certain flowers that have a horrible smell*. Women reported that abortions were common and could be done regardless of how advanced the pregnancy was. Abortion was viewed as a simple process where *all you need is for the womb to open, then the baby will be expelled immediately*.

### Confidence levels and correct contraceptive usage

Survey respondents expressed high levels of confidence in their ability to correctly use their chosen contraceptive method(s): 85.8% (*n* = 205) were “very confident” or “extremely confident”, while respondents from LMIC reported less frequently that they felt “extremely confident” (43.0% vs 67.4%; *p* = 0.001).

Several respondents felt less confident, however, in the abilities of their partners (including clients and other sexual partners).

“*[I feel] fairly confident in my own ability to use [condoms] correctly. I feel less confident in all of the variables surrounding using a condom with a partner.*” (IDI participant, Americas)

Several IDI participants expressed concern about using intrauterine devices (IUDs) while having multiple partners. Some were worried that this would cause the device to be “moved” or “pushed”, causing pain and potentially reducing efficacy.

“*With the coil, I wouldn’t say I’m confident because sometimes when you have so many clients, you feel sometimes as though the coil has been pushed.*” (IDI participant, Africa)

“*The loop is not the method that female sex workers prefer to use because of multiple partners and the fear that at any time it can be touched, moved, or swung out of place … because of the nature of the work that we are doing. [We need] more information about it and how it works, especially for a person with multiple partners.*” (IDI participant, Africa)

FGD participants reported that insertion of female condoms required skill that many did not possess.

“*It’s hard to correctly position the ring of the female condom. He will say he is in pain, and you will also be in pain. So, you end up doing the wet one [condomless sex].*” (24-year-old woman, FGD)

### Preferred contraceptive attributes and dosing regimens

Of online survey respondents, 87% (*n* = 207) felt that preventing pregnancy within the next three years was “extremely important” or “very important”. Ease of access (67.4%, *n* = 161), efficacy in pregnancy prevention (66.9%, *n* = 160), ease of use (60.7%, *n* = 145), protection against HIV and STIs (55.6%, *n* = 133) and minimal side effects (54.4%, *n* = 130) were contraceptive attributes identified as “extremely important”. Less important were recommendations made by healthcare providers (33.9%, *n* = 81) or personal acquaintances (36.4%, *n* = 87).

“*Something without side effects. Reliability. Ease of use. Those would be the three things [most] important to me. It really just has to do with being able to lead a normal life and a normal sex life without too many complicating factors.*” (IDI participant, Americas)

**Figure 1.**
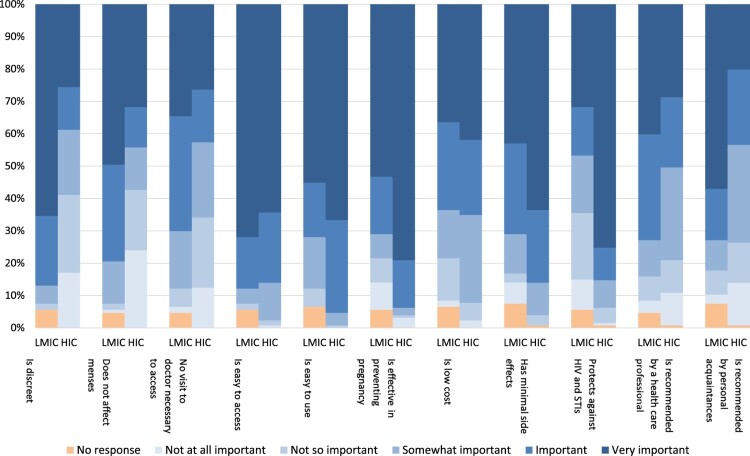
Importance of contraceptive attributes, comparing respondents from high-income countries (HIC) and low- and middle-income countries (LMIC)

HIC and LMIC participants had different views on contraceptive attributes. Respondents from LMIC more frequently rated as “extremely important” that the method was discreet (65.4% vs 25.6%), did not affect menses (49.5% vs 31.8%), was recommended by acquaintances (57.0% vs 20.2%), was recommended by healthcare professionals (40.2% vs 28.7%), and was easy to access (72.0% vs 64.3%). Those from HIC more frequently rated as “extremely important” that the method was easy to use (66.7% vs 55.1%), prevents pregnancy (79.1% vs 53.3%), had minimal side effects (63.6% vs 43.0%), and protects against HIV and STIs (75.2% vs 31.8%) (Figure 1).

IDI participants differed in their preferences for certain attributes, for example, with regard to contraceptive methods that affect menstruation.

“*Feeling my menstrual cycle matters in my choice (copper IUD) [whereas] hormonal stuff kills it. And I love my cycle – it’s not only about menses.*” (IDI respondent, Europe)

“*Currently I don’t have periods, which I prefer. Anything causing me to have a period I don’t wanna be on.*” (IDI respondent, Americas)

Vulnerability to violence is another determinant of contraceptive values and preferences. Several IDI participants expressed a preference for discreet dual contraception due to difficulties negotiating condom use with violent or aggressive clients, or for protection in the event of rape.

“* … * *The one advantage for the coil is that you can’t easily get pregnant. And … it’s inside, so the client wouldn’t be able to tell that you have a coil* * … * *And maybe you get someone who is violent and who forces you to have sex without a condom, so for me it’s been very good to have both of them.*” (IDI participant, Africa)

An important component of access was cost: FGD participants said it was critical to get contraceptives for free.

“*To be honest things are hard at the moment. Most clinics require you to pay for services, but we just don’t have the money.*” (36-year-old woman, FGD)

FGD participants complained about bleeding/spotting with depot medroxyprogesterone acetate (DMPA) or implants as it interferes with sex work. There were also concerns about weight gain with DMPA and implants, although women’s views on this were mixed, with some reporting that weight gain was good for business as they believed many men preferred big women. Infertility was considered a worrisome side effect of both methods, with many believing that the infertility was permanent.

In FGDs the pill was considered the method with fewest side effects, although the daily dosing is difficult.

“*In my view pills are not ideal for us sex workers. But sometimes they are the only ones which don’t cause side effects … If only a pill could be made that you only have to take once a year.*” (34-year-old woman, FGD)

“*With loop you can be in constant pain that is similar to period pain. That will keep disturbing my work.*” (38-year-old woman, FGD)

A recurrent view among IDI participants was the need for a method that did not require daily or weekly use. Participants’ everyday activities such as unexpected travel with a client could cause adherence problems.

“* … * *But I had to take it* (the pill) *every day, and I’m a little forgetful. So, while I was able to keep up with the doses, I didn’t really trust myself long-term with it, so that’s when I decided to get an IUD.*” (IDI participant, Western Pacific)

“*I actually really like that it* (DMPA) *[lasts] 3 months, and I just do it and forget about it* * … * *I do sex work in different countries, and I don’t have a 9-to-5 lifestyle or a really regular schedule.*” (IDI participant, Europe)

Preferred dosing regimens also vary greatly. Survey respondents expressed a preference for methods that are coitus-dependent (30.5%, *n* = 73), long-acting (27.6%, *n* = 66), or permanent (20.9%, *n* = 50). However, in LMIC compared to HIC, more women preferred methods that were taken daily (12.1% vs 3.9%) or every few months (14% vs 9%), Table 3.

### Perception of HIV risk and its impact on contraceptive decision-making

Over two-thirds of survey respondents (69.45%, *n* = 166) reported HIV and STI prevention as either an extremely or very important contraceptive attribute. Nonetheless, 42.7% (*n* = 102) of them perceived their own HIV risk as being low, and 10.0% (*n* = 24) perceived their risk as being non-existent. Compared to women from HIC, women from LMIC more frequently perceived their HIV risk to be “very high” (8.4% vs 3.9%) or “high” (15.0% vs 3.9%) (Table 4).

**Table 3.** Preferred dosing

		Low- and middle-income countries (*n* = 107)	High-income countries (*n* = 129)	*χ*^2^*p*-value	TOTAL (*n* = 239)^a^
**Prefer a contraceptive method that:**	Take every day	13 (12.1%)	5 (3.9%)	0.007	19 (7.9%)
	Take every few months	15 (14.0%)	9 (7.0%)		24 (10.0%)
	Take every few weeks	3 (2.8%)	2 (1.6%)		5 (2.1%)
	Use only when having sex	36 (33.6%)	35 (27.1%)		73 (30.5%)
	Lasts forever	13 (12.1%)	37 (28.7%)		50 (20.9%)
	Protects against pregnancy for years	26 (24.3%)	40 (31.0%)		66 (27.6%)
	No response	1 (0.9%)	1 (0.8%)		2 (0.8%)

^a^ Includes 3 respondents not reporting country of residence and omitted from analysis of country income categories.

**Table 4.** Perception of risk and importance of pregnancy, HIV and STI prevention

		Low- and middle-income countries (*n* = 107)	High-income countries (*n* = 129)	*χ*^2^*p*-value	TOTAL (*n* = 239)^a^
How important: Effective prevention of pregnancy	Extremely	57 (53.3%)	102 (79.1%)	0.000	160 (66.9%)
	Very	19 (17.8%)	19 (14.7%)		40 (16.7%)
	Somewhat	8 (7.5%)	3 (2.3%)		11 (4.6%)
	Not so	8 (7.5%)	1 (0.8%)		9 (3.8%)
	Not at all	9 (8.4%)	4 (3.1%)		13 (5.4%)
	No response	6 (5.6%)	0 (0.0%)		6 (2.5%)
How important that it protects against HIV and STIs	Extremely	34 (31.8%)	97 (75.2%)	0.000	133 (55.6%)
	Very	16 (15.0%)	13 (10.1%)		30 (12.6%)
	Somewhat	19 (17.8%)	11 (8.5%)		30 (12.6%)
	Not so	22 (20.6%)	6 (4.7%)		28 (11.7%)
	Not at all	10 (9.3%)	1 (0.8%)		11 (4.6%)
	No response	6 (5.6%)	1 (0.8%)		7 (2.9%)
Perception of own HIV risk	Very high risk	9 (8.4%)	5 (3.9%)	0.002	14 (5.9%)
	High risk	16 (15.0%)	5 (3.9%)		22 (9.2%)
	Moderate risk	26 (24.3%)	38 (29.5%)		66 (27.6%)
	Low risk	34 (31.8%)	68 (52.7%)		102 (42.7%)
	No risk	16 (15.0%)	8 (6.2%)		24 (10.0%)
	No response	1 (0.9%)	1 (0.8%)		2 (0.8%)

^a^ Includes 3 respondents not reporting country of residence and omitted from analysis of country income categories.

IDI participants’ perceptions of their own HIV risk typically reflected insight into their local epidemic context. Those in the WHO Africa region conveyed greater concern about the local HIV epidemic and perceived their own risk as being high. This concern extended to the well-being of their peers and communities.

“*With female sex workers and our practices, we are concerned that we have a higher prevalence of HIV than among the general population. In [our country], for female sex workers, the prevalence is 26%, when the general population is 4.7%, so that’s why we are aware of that, and also we are concerned about it.*” (IDI participant, Africa)

“*[My HIV risk is] very high, about 80%. Because in a week, maybe I have about 10 men that I’m selling sex to.*” (IDI participant, Africa)

IDI participants in low-prevalence countries tended to perceive their own HIV risk as being low, while expressing varying levels of concern surrounding the virus.

“*HIV, I’m not concerned about in the slightest. It’s the first test that I get back … * *and I get the text message, and I don’t even like to look at it. It’s like, ‘Oh yeah, it’s negative.’ Within straight populations in Australia it is almost non-existent, amongst people that aren’t IV drug users. So, it is quite difficult to catch if you’re not having bareback anal sex, if you’re not having sex with high-risk people. So, I’m not really in the risk category for it.*” (IDI participant, Western Pacific)

Other IDI participants acknowledged their potential risk of acquiring HIV but felt reassured by the availability of prevention and treatment options.

“*[I am] a little bit concerned, because I know I have a higher risk than other populations, but there are treatments for it now and I can also make the choice to use PrEP, which I haven’t decided to do since I’m afraid of side effects. But I feel a little bit more confident that I can have a life if I were to contract it, or that there are ways that I can keep myself from contracting it.*” (IDI participant, Americas)

IDI participants invariably emphasised the importance of consistent condom use for preventing HIV, and two out of 12 interview participants reported current use of PrEP. Many respondents also reported regular HIV testing to monitor their status. One respondent additionally described their role in counselling peers on HIV prevention.

“*I’ve [educated myself] about how [HIV] is transmitted, what my risks are, and what to do if there was an emergency. I find myself constantly counselling colleagues who aren’t on PrEP on how they can go and access PEP [post-exposure prophylaxis] if they’re in a situation where they decide that’s necessary.*” (IDI participant, Europe)

Given the limited accessibility to PrEP in many regions of the world, condoms remain a highly valued contraceptive method due to their dual-protection properties.

### The role of other people in contraceptive decisions

Survey results indicated that healthcare providers were most likely to influence contraceptive decisions with respondents reporting that they were “very likely” (36.71%, *n* = 87) or “somewhat likely” (38.9%, *n* = 93) to influence their contraceptive decisions; this was consistent across HIC and LMIC respondents (Table 5).

**Table 5.** Influence of others on contraceptive decision-making

		Low- and middle-income countries (*n* = 107)	High-income countries (*n* = 129)	*χ*^2^ *p*-value	TOTAL (*n* = 239)^a^
**Healthcare professional likely to influence contraceptive decisions**	Very likely	52 (48.6%)	35 (27.1%)	0.010	88 (36.8%)
	Somewhat likely	31 (29.0%)	60 (46.5%)		93 (38.9%)
	Neither likely nor unlikely	9 (8.4%)	13 (10.1%)		22 (9.2%)
	Somewhat unlikely	6 (5.6%)	9 (7.0%)		15 (6.3%)
	Very unlikely	4 (3.7%)	10 (7.8%)		14 (5.9%)
	N/A	3 (2.8%)	2 (1.6%)		5 (2.1%)
	No response	2 (1.9%)	0 (0.0%)		2 (0.8%)
**Clients likely to influence contraceptive decisions**	Very likely	15 (14.0%)	0 (0.0%)	<0.001	16 (6.7%)
	Somewhat likely	17 (15.9%)	12 (9.3%)		29 (12.1%)
	Neither likely nor unlikely	9 (8.4%)	16 (12.4%)		26 (10.9%)
	Somewhat unlikely	9 (8.4%)	13 (10.1%)		22 (9.2%)
	Very unlikely	30 (28.0%)	77 (59.7%)		108 (45.2%)
	N/A	13 (12.1%)	9 (7.0%)		22 (9.2%)
	No response	14 (13.1%)	2 (1.6%)		16 (6.7%)
**Spouse likely to influence contraceptive decisions**	Very likely	26 (24.3%)	14 (10.9%)	<0.001	41 (17.2%)
	Somewhat likely	28 (26.2%)	27 (20.9%)		56 (23.4%)
	Neither likely nor unlikely	13 (12.1%)	20 (15.5%)		33 (13.8%)
	Somewhat unlikely	7 (6.5%)	5 (3.9%)		12 (5.0%)
	Very unlikely	26 (24.3%)	14 (10.9%)		32 (13.4%)
	N/A	9 (8.4%)	41 (31.8%)		51 (21.3%)
	No response	13 (12.1%)	1 (0.8%)		14 (5.9%)
**Other sexual partners likely to influence contraceptive decisions**	Very likely	17 (15.9%)	6 (4.7%)	<0.001	24 (10.0%)
	Somewhat likely	16 (15.0%)	26 (20.2%)		43 (18.0%)
	Neither likely nor unlikely	15 (14.0%)	25 (19.4%)		41 (17.2%)
	Somewhat unlikely	16 (15.0%)	26 (20.2%)		28 (11.7%)
	Very unlikely	20 (18.7%)	37 (28.7%)		57 (23.8%)
	N/A	14 (13.1%)	19 (14.7%)		33 (13.8%)
	No response	13 (12.1%)	0 (0.0%)		13 (5.4%)
**Peers likely to influence contraceptive decisions**	Very likely	24 (22.4%)	16 (12.4%)	<0.001	41 (17.2%)
	Somewhat likely	29 (27.1%)	51 (39.5%)		82 (34.3%)
	Neither likely nor unlikely	12 (11.2%)	26 (20.2%)		38 (15.9%)
	Somewhat unlikely	8 (7.5%)	13 (10.1%)		21 (8.8%)
	Very unlikely	13 (12.1%)	16 (12.4%)		29 (12.1%)
	N/A	7 (6.5%)	7 (5.4%)		14 (5.9%)
	No response	14 (13.1%)	0 (0.0%)		14 (5.9%)

^a^ Includes 3 respondents not reporting country of residence and omitted from analysis of country income categories.

In the absence of comprehensive contraceptive counselling from healthcare providers, one IDI respondent described the vital role of peer-to-peer education in informing sex workers’ contraceptive decisions. This is also reflected in survey results where 17.2% (*n* = 41) and 34.3% (*N* = 82) responded that peers were respectively “very likely” and “somewhat likely” to influence contraceptive decision-making (Table 5).

“*Peer-to-peer education plays a very big role because that’s where we learn from each other [about] the best contraception, and* * … * *side effects.*” (IDI participant, Africa)

Overall, the influence of clients on contraceptive decisions was rated “very unlikely” by 45.2% (*n* = 108) of survey respondents, although this differed depending on setting with 59.7% (*n* = 77) of respondents from HIC responding “very unlikely” compared to 28% (*n* = 30) from LMIC (Table 5). Qualitative data confirmed this difference by setting; FGD participants reported that clients could influence the choice of contraceptive method, including the decision to stop using it, while participants from other settings did not allow clients to have influence:

“*He will say a woman on jadelle is not ‘delicious’ because it blocks the womb and prevents it from producing the sweetness that causes us to enjoy each other.*” (19-year-old woman, FGD)

“*When it comes to clients, it’s not negotiable. They know what services I use condoms for, and what I don’t use condoms for … * *And everything’s kind of set out, you know? You pay for [the services] and it’s not negotiable.*” (IDI participant, Western Pacific)

Clients who are violent or aggressive may affect sex workers’ ability to consistently use contraception, and clients who offer higher payment for condomless sex may also influence some sex workers’ willingness or ability to consistently use condoms. Several respondents noted that using a second, covert method of contraception engendered a greater sense of control and autonomy.

“*With Depo, I know it’s something that is in me, it’s something that I cannot negotiate with someone* * … * *it’s something that cannot be touched* * … * *With condoms, it’s something that I have to wear, or the man has to wear, so it becomes a challenge. But with Depo* * … * *I feel like I am in total control.*” (IDI participant, Africa)

### Coerced childbearing

FGD results revealed that clients sometimes requested/expected sex workers to have children with them. A client’s request for a child reportedly placed significant pressure on sex workers because of financial vulnerability. FGD participants said openly refusing to have the requested child would end the relationship and the financial support. In such instances they would have unprotected sex and were not expected (by the clients) to be using contraception, hence the need for discreet methods. A recurrent view among FGD participants was that sometimes clients were unhappy when they discovered that women were, in fact, using contraception. Participants suspected that many of these men would try to alter their contraceptive methods (rendering them ineffective) in order to assure pregnancy, for example subjecting oral contraceptives to extreme heat, or tampering with implant rods.

“*Most of us in this sex work business have those whom we call our permanent boyfriends, the ‘I love you’s, those who deceive us. He will say, ‘Baby, if you only bear a child for me the heavens would have opened for me’.*” (Woman aged over 25 years [NB participant preferred not to share age], FGD)

“*They want children. One can take your pills and boil them. By the time you take the pills they have no power and you find yourself pregnant.*” (33-year-old woman, FGD)

### Causes of contraceptive switching and/or discontinuation

Numerous factors may affect individuals’ ability or willingness to continue using a contraceptive method. Survey respondents indicated a wide range of situations, outcomes, and side effects which would cause them to switch or discontinue their current contraceptive method(s). Increased risk of STIs (71. 5%, *n* = 171) and HIV (68.2%, *n* = 163), increased bleeding (66.1%, *n* = 158), nausea/vomiting (64.0%, *n* = 153), and unintended pregnancy while using the method (64.0%, *n* = 153) were mentioned as situations most likely to cause contraceptive switching or discontinuation. Increased risk of HIV was more likely to be reported as a reason for cessation or discontinuation by women living in HIC vs LMIC (83.7% vs 49.5%; *p* < 0.0001), as was increased risk of STIs (86.0% vs 54.2%; *p* < 0.0001) (Table 6). One IDI participant with access to affordable abortion services explained that while an unplanned pregnancy could be terminated, certain STIs could not be cured.

“*Pregnancy, to me, is something that is less of a concern than a disease, because pregnancy is fixable. I’m pro-abortion. I’d prefer not to have to get an abortion, but it’s not my biggest concern in the world.*” (IDI participant, Western Pacific)

Disapproval from clients was more frequently reported as a reason for cessation or discontinuation of use by women from LMIC vs HIC (26.2% vs 10.9%; *p* = 0.002), as was disapproval from a spouse (19.6% vs 10.1%; *p* = 0.038), but disapproval from other sexual partners as a reason was consistently low in both LMIC and HIC, Table 6.

**Table 6:** . Causes of contraceptive switching and/or discontinuation

		Low- and middle-income countries (*n* = 107)	High-income countries (*n* = 129)	*χ*^2^ *p*-value	TOTAL (*n* = 239)
**What would cause cessation or discontinuation of contraceptive use**	Difficulties accessing methods	41 (38.3%)	84 (65.1%)	<0.001	127 (53.1%)
	Disapproval from clients	28 (26.2%)	14 (10.9%)	0.002	42 (17.6%)
	Disapproval from spouse	21 (19.6%)	13 (10.1%)	0.038	34 (14.2%)
	Disapproval from other sexual partners	13 (12.1%)	9 (7.0%)	0.174	22 (9.2%)
	Disruptions to menstrual cycle	50 (46.7%)	50 (38.8%)	0.217	102 (42.7%)
	Dizziness	41 (38.3%)	81 (62.8%)	<0.001	124 (51.9%)
	Increased bleeding	61 (57.0%)	95 (73.6%)	0.007	158 (66.1%)
	Increased risk of HIV from use	53 (49.5%)	108 (83.7%)	<0.001	163 (68.2%)
	Increased risk of STIs from use	58 (54.2%)	111 (86.0%)	<0.001	171 (71.5%)
	Inconvenient to use	47 (43.9%)	77 (59.7%)	0.016	126 (52.7%)
	Too expensive	34 (31.8%)	87 (67.4%)	<0.001	123 (51.5%)
	Nausea/vomiting	50 (46.7%)	101 (78.3%)	<0.001	153 (64.0%)
	Unintended pregnancy while using	50 (46.7%)	101 (78.3%)	<0.001	153 (64.0%)
	Weight gain or weight loss	43 (40.2%)	75 (58.1%)	0.006	120 (50.2%)
	Other	2 (1.9%)	21 (16.3%)	-	23 (9.6%)

^a^ Includes 3 respondents not reporting country of residence and omitted from analysis of country income categories.

Several IDI respondents expressed concern about the effects of hormonal contraception on short- and long-term health.

“*I’d taken [the pill] continuously for about 7 or 8 years* * … * *With age, I had become concerned that it could be causing health effects. I had my uterine lining scanned, and everything’s fine* * … * *But I thought maybe, because I have very thin hair, maybe it’s affecting my hair. Or maybe it’s affecting some other part of my system in a very slight way, so I just wanted to go off it to see how I felt.*” (IDI participant, Western Pacific)

### Other barriers to accessing contraceptives

Most survey respondents reported no barriers to access contraception, and this was consistent across high- and low-income settings (61.7% in LMIC and 54.3% in HIC). The most frequently reported barrier was high cost (23.5%; *n* = 56), although less so in LMIC (18.7% vs 27.9%; *p* = 0.002). Women in HIC more frequently reported discrimination (20.9% vs 9.3%; *p* = 0.015) and refusal of services by healthcare providers (11.6% vs 3.7%; *p* = 0.027). Sex workers in FGD and IDI reported that the provision of services in a non-judgemental way was critical to uptake of services.

“*There is no privacy when you collect condoms, and they [nurses] glare at you and ask if you will use all those condoms.*” (Woman in FGD group that did not access Sisters services [NB participant preferred not to disclose her age])

“*Sometimes in public services it is hard to get contraceptives, because they take you as someone who is sinful. They can’t receive you well, being a sex worker. They say, ‘You don’t have a husband. How can you be accessing contraception?’*” (IDI participant, Africa)

Stockouts reduce access to contraceptives. FGD participants complained about DMPA supply shortages at public sector clinics. Women also noted that bulk packaging was a barrier; to save stocks, nurses were only willing to open the package if they knew they would use all the product. Some clinics had specific “family planning days”; if an individual visited the clinic on a wrong day, then they would not get the service.

“*Sometimes when you go to the clinic [to get] the depot injection, they can tell you that [they are] not opening the medicine vial unless there are five people, because the vial is for five people. So you need to wait until there are five people, [and] if five people don’t come they tell you to go back home and come back another day.*” (40-year-old woman, FGD)

FGD and IDI participants reported that they were often forced into choices by healthcare workers. For example, young women who wanted DMPA or implants were discouraged with reasons that these methods could cause infertility, while women on ART were discouraged from using implants because of drug interactions. The IUD was not recommended for women with many partners.

“*If you are on ART and use jadelle they* (health workers) *will tell you to remove it whether you like it or not because the ART medicine will overpower jadelle and … you might get pregnant.*” (24-year-old woman, FGD)

“*Loop (IUD) should only be used by women with few partners, such as married women who may only have one other permanent partner that they see once a month. But it has been said that loop is not recommended for women with seven or eight partners a day because it can shift.*” (38-year-old woman, FGD)

## Discussion

This mixed-methods study among sex workers examined values and preferences related to contraception in different income settings. The survey revealed overall good knowledge of contraceptive methods. However, when explored in more depth misconceptions were common. Barriers to access and use of contraceptives were more common in LMIC online survey participants; this is consistent with findings from other studies in LMIC.^16^ One in five women said they had used emergency contraception and unplanned pregnancies were common, which indicates unmet contraceptive needs. Abortion was reported in FGDs as sometimes unsafe and there were misconceptions about abortion methods. Influence on contraceptive decision-making differed depending on where sex workers lived.

In LMIC, online survey participants placed greater value on the preferences and influences of clients and spouses than those in HIC. In Zimbabwe FGDs, women reported pressure from clients to engage in unprotected sex because of increased pleasure and in some instances their clients’ desire for children. The notion of male dominance in sex workers’ relationships has also been reported in India, where it is reportedly accepted by sex workers in an attempt to make the relationship more akin to marital relationships which are thought of as more socially acceptable.^30^ This desire for social acceptability may limit women’s willingness to fight for their rights to contraceptive choices. This and other cultural beliefs and social norms need to be taken into account when designing programmes for contraception that optimise uptake by all groups.^31^ Importantly, programmes that optimise uptake of contraception may simultaneously improve sex workers’ agency to make decisions about other interventions, such as HIV testing and PrEP. Pressure from clients and the inability to negotiate condom use may be more difficult for younger sex workers.^32^ Women in Zimbabwe FGDs reported fear of loss of income if they refused to engage in sex without protection/contraceptives. Related, women in LMIC participating in the online survey were also more likely to value contraceptives that are discreet, so that they did not need to disclose to clients that they were avoiding pregnancy or to negotiate condom use.

It is also critical that male partners, especially in LMIC, are engaged/educated to appreciate the importance of safer sex. It may also be important to dispel the myths among men surrounding the use of certain contraceptives, in order to remove the pressure that men place on women to stop using these methods. Sex worker empowerment efforts should continue so that contraceptive users have the skills to negotiate safer sex and use of contraception without fear of loss of income. Peers were valued for their contraceptive advice and could have a greater role.

As documented elsewhere, sex workers reported more frequent condom use with clients than with other sexual partners,^33^ highlighting the need for information and counselling to support safer sex practices with non-client sexual partners and to make HIV testing and partner-testing services more easily available to inform HIV prevention choices.

Sex workers reported relying on information from healthcare providers when making contraceptive decisions and placed a high value on this information. At the same time, judgemental attitudes from healthcare providers created a barrier to sex workers accessing contraceptives. This is a recurring barrier affecting sex workers and has been reported in many settings.^34–36^ Healthcare providers have an important role in ensuring sex workers can make informed choices about contraception. The ongoing unmet contraception need is a critical issue for sex workers and efforts should be made to increase access by making services more accessible and available at times and places convenient for them. It is important that health workers are trained to provide correct information that is in line with recommendations/guidelines, and efforts should be made to ensure stigma-free health services for sex workers.

Women expressed a wide variation in values and preferences related to contraception and their preferences for specific contraceptive methods. None of the available contraceptive methods meet all the criteria of a “preferred product”. For example, while DMPA has the advantage of not requiring daily use and is discreet and effective in preventing pregnancy, for some women it has unfavourable side effects and health system barriers exist when trying to access. In different settings, contraceptive preferences and use varied, indicating that WHO and other global bodies must ensure promotion and guidance related to a wide range of contraceptive methods. This also highlights a need for choice and person-centred care and advice on when and where to provide contraceptive services for sex workers.

Dual protection – condom use alongside reliable modern contraception – has long been promoted as essential for the prevention of unplanned pregnancies and HIV prevention.^37^ This requires the availability of a range of contraceptives as well as condoms and lubricant. However, condom supplies for sex workers are inadequate. In Zimbabwe, for example, a case study reported that although there is significant funding for condom programmes, limited coordination between government and funders may be responsible for some gaps.^38^ Globally there has been a decline in the emphasis on condoms and a reduction in funding for these programmes.^39^ A reinvigoration of condom programming is critical, including addressing barriers to access for sex workers, while also recognising the need for a coordinated approach to the provision of family planning which would ensure the availability of a range of methods in response to the diversity in women’s contraceptive preferences. This could include more information and support for the use of female condoms, as challenges with use, including insertion, are reported.^40,41^

This mixed-methods study enabled participation through the online survey of sex workers in different global regions and more in-depth discussion of issues in the FGDs and IDIs. There were a number of limitations to the study. The survey was not a representative sample, hence findings may not be generalisable. However, with support of the Global Network of Sex Work Projects, it was widely advertised on various media to promote participation by geographically, linguistically, and socio-economically diverse groups. Although participants were not required to disclose their gender identity beyond confirming their eligibility, the survey and IDIs were deliberately designed to promote the inclusion of sex workers with diverse gender identities. The survey only reached those with access to the internet and with good literacy, again limiting generalisability. This may also lead to bias as those that do not have internet access may be those that are more marginalised, meaning it is possible that there was an under-representation of this group. The FGD findings are also not generalisable; for example, the models of sex worker operation/reach, legal framework and national programmes that target sex workers in Zimbabwe are likely to be significantly different from other settings. All findings were also based on self-reporting. Despite these limitations, this study provides some important insights into the values and preferences for contraceptives for sex workers. This is the first time WHO has specifically included their voices as part of developing global contraceptive guidance.

In conclusion, although in our study sex workers have good awareness of contraceptives, this does not translate into good access, choice, and use. Barriers that include partners/clients and health system factors need to be addressed as we strive for universal health coverage which leaves no one behind.

## Supplementary Material

Supplement_1_Online_survey_questionnaire
