# Supplementary material for: Values and preferences of contraceptive methods: a mixed-methods study among sex workers from diverse settings
Source: Sex Reprod Health Matters. 2021 May 5;29(1):1913787. doi: 10.1080/26410397.2021.1913787 (PMC8118510; doi:10.1080/26410397.2021.1913787)
Supplement: Supplement_1_Online_survey_questionnaire [file ZRHM_A_1913787_SM7518.docx]

| **Supplement 1: Online survey questionnaire**  **Female Sex Workers’ Practices, Values, and**  **Preferences Regarding Contraception** |
| --- |

Participant Information and Informed Consent Form

The World Health Organization (WHO) Department of HIV, Key Populations and Innovative Prevention Team, is conducting a study of female sex workers’ values and preferences regarding contraception, as well as their experiences using different contraceptive methods. Please read the following information before deciding whether you would like to participate in this research. 
 
**Purpose and Scope of Research**
WHO would like to learn more about sex workers’ experiences with and attitudes towards contraception. This survey seeks information on which contraceptive methods sex workers use, how often they use them, and which methods they know of. This survey also seeks to learn more about sex workers’ motivations and preferences when choosing different contraceptive methods, as well as any barriers they may face. Knowing this information will help WHO improve its guidance materials on the topic of contraception, so that health care providers around the world can better serve the sexual and reproductive health needs of sex workers.  
 
This survey contains 23 questions and will take approximately 15 – 20 minutes of your time. Your participation is entirely voluntary, and all questions, with the exception of the consent and eligibility screening questions, are optional and may be skipped. You do not need to answer every question in order to complete the survey. If you choose to participate but then change your mind, you are free to exit the survey at any time. If you would like to change your responses, you may do so before submitting the survey. 
 
**Eligibility**
In order to participate in this survey, you must meet the following criteria:
·      **Female, or gender variant individual capable of giving birth**
·      **Current sex worker**
·      **Aged 18-49**
 
**Confidentiality**
All survey responses are sent over a secure, encrypted connection. Any information you provide is anonymous and cannot be traced back to the individual. We will not ask for any names or other identifying information, and your IP address will not be tracked. Anonymous survey data will be exported to a password protected computer that is only accessible to the researcher. All data will be deleted upon completion of the project in August, 2019.
 
**Use of Data** 
Findings from this survey will be shared at the WHO Guidelines Development Group meeting in Geneva in July, 2019. Following the meeting, the research findings will be used to create a written report for publication.
 
**Benefits and Risks**
We are asking you to share some personal and confidential information with us, and you may feel uncomfortable answering some of the questions. While there will be no direct incentive or benefit to you, your participation in this survey will help us learn more about your community’s needs and preferences in the realm of contraception. This proposal has been reviewed and approved by the WHO Ethics Review Committee, which is a committee whose task it is to make sure that research participants are protected from harm.

**Contact**

If you have any questions or concerns, or if you would like to discuss this topic further, please contact Virginia Macdonald at macdonaldv@who.int. You may also leave any comments or questions you may have at the end of the survey.

1. **Consent and eligibility questions**


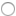
 I have read and understood the information sheet and consent to participate in this research


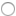
 I am female (or gender variant), between the ages of 15 to 49 and am currently engaged in sex work

1. **What year were you born?** ___________________
2. **How many children do you have?** ___________________
3. How important is it for you to avoid pregnancy within the next 3 years?


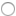
 Extremely important


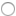
 Very important


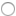
 Somewhat important


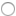
 Not so important


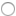
 Not at all important


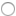
 Unsure

1. **What is your country of residence?** ___________________
2. **What is the highest level of education you have completed?**


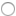
 No formal education


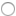
 Primary school


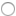
 High school


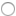
 Advanced education


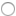
 University or higher

1. **Where do you access information on contraception? Please check all that apply.**


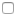
 At or through work


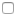
 Drop-in centers


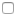
 Friends or family


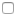
 Health care providers


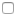
 Mobile clinics


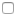
 Non-governmental or community-based organizations


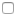
 Outreach workers


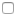
 The Internet


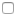
 Other: ___________________


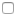
 I don’t access any information on contraception

1. **Which contraceptive methods have you heard of? Please check all that apply.**

Top of Form

| **Non-Hormonal Methods** | **Hormonal Methods** |
| --- | --- |
| 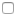 Male condoms | 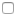Oral hormonal contraceptive pills (“the pill”) |
| 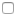Female condoms | 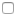Vaginal contraceptive rings |
| 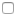Diaphragms/cervical caps | 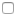Hormonal contraceptive patches |
| 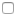Non-hormonal (copper) intrauterine devices | 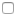Hormonal intrauterine devices |
| 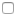Tubal litigation (female sterilization) | 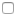Injectable hormonal contraceptives |
| 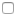Vasectomy (male sterilization) | 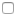Hormonal contraceptive implants |
| 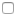Traditional contraceptive methods (rhythm, withdrawal, etc.) | 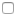Emergency contraception (“Morning after pill” that can be taken after unprotected sex) |

Bottom of Form

1. **Which contraceptive methods have you used within the last year? Please check all that apply.**

| **Non-Hormonal Methods** | **Hormonal Methods** |
| --- | --- |
| 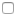 Male condoms | 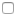Oral hormonal contraceptive pills (“the pill”) |
| 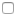Female condoms | 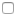Vaginal contraceptive rings |
| 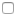Diaphragms/cervical caps | 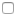Hormonal contraceptive patches |
| 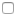Non-hormonal (copper) intrauterine devices | 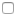Hormonal intrauterine devices |
| 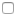Tubal litigation/ (female sterilization) | 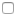Injectable hormonal contraceptives |
| 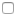Vasectomy (male sterilization) | 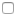Hormonal contraceptive implants |
| 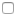Traditional methods (rhythm, withdrawal, etc.) | 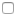Emergency contraception (“Morning after pill” that can be taken after unprotected sex) |

1. **How regularly do you use condoms:**

With clients: With other male partners:

All of the time
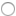

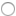


More than half of the time
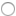

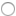


Half of the time
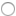

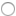


Less than half of the time
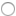

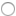


Never
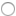

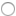


Not applicable
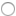

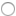


1. **How regularly do you use other contraceptive methods:**

With clients: With other male partners:

All of the time
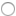

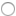


More than half of the time
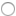

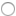


Half of the time
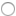

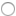


Less than half of the time
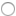

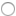


Never
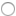

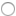


Not applicable
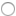

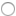


Bottom of Form

1. **How confident do you feel in your ability to correctly use your chosen contraceptive method(s)?**


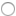
 Extremely confident


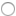
 Very confident


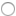
 Somewhat confident


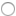
 Not so confident


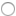
 Not at all confident


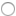
 I do not use any contraceptive methods

1. **How satisfied are you with your current contraceptive method(s)?**


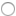
 Very satisfied


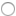
 Satisfied


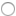
 Neither satisfied nor dissatisfied


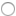
 Dissatisfied


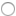
 Very dissatisfied


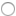
 I do not use any contraceptive methods

1. **Do you feel like your contraceptive needs are being met?**


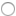
 Yes


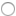
 No.


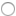
 Not sure.


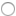
Other (please explain)

1. **How important are the following qualities to you when choosing a contraceptive method?**

Not at all important Very Important

Discreet 0 1 2 3 4 5

Does not affect normal menses 0 1 2 3 4 5

Does not require visit to the doctor 0 1 2 3 4 5

Easy to access 0 1 2 3 4 5

Easy to use 0 1 2 3 4 5

Effective at preventing pregnancy 0 1 2 3 4 5

Low cost / affordability 0 1 2 3 4 5

Minimal side effects 0 1 2 3 4 5

Protects against HIV infection / STIs 0 1 2 3 4 5

Recommended by healthcare providers 0 1 2 3 4 5

Recommended by personal acquaintances 0 1 2 3 4 5

1. How high or low do you perceive your HIV risk to be?


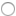
 Very high risk


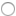
 High risk


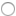
 Moderate risk


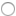
 Low risk


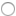
 No risk


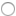
 Not sure / not applicable

1. **I would prefer a contraceptive method that:**


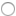
 I take every day


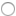
 I take every few weeks


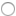
 I take every few months

Protects me against pregnancy for years

Lasts forever

I use only when I have sex

TVop of Form

Bottom of Form

1. **How likely are the following people to influence your contraceptive decisions?**

Not at all likely Extremely likely

Health Care Providers 0 1 2 3 4 5

Clients 0 1 2 3 4 5

Spouse 0 1 2 3 4 5

Other Sexual Partners 0 1 2 3 4 5

Peers 0 1 2 3 4 5

1. **What situations would cause you to discontinue or switch your contraceptive method(s)? Please check all that apply.**

Difficulties accessing method

- Disapproval from clients
- Disapproval from spouse

Disapproval from other sexual partners

Disruptions in menstrual cycle

Dizziness

Increased bleeding

Increased risk of HIV from use

Increased risk of STIs from use

Method is inconvenient to use

Method is too expensive

Nausea / vomiting

Unintended pregnancy while using

Weight gain or weight loss

Other: _________________

1. **Where do you typically access contraception? Please check all that apply.**

At or through work

Directly from the pharmacy

Drop-in centers

Family planning clinic

Friends or family members

Mobile clinics

Non-governmental or community organizations

Outreach workers

Private clinic or hospital

Public clinic or hospital

Other: _____________

1. **Do you face any barriers to accessing contraception? If so, please check all that apply.**

I do not face any barriers / not applicable

Discrimination / poor treatment from health care providers

Distant and/or inconvenient locations

High costs

Inconvenient opening hours

Lack of available supplies (stock-outs)

Long waiting times

Refusal of service by health care providers

Risks of legal repercussions due to being a sex worker

Other: ___________________

1. **Was language a barrier for you in completing this survey?**

**Yes**

- **No**
- **Not sure (please explain)**

1. **Do you have anything else you would like to share or comment on?** ___________________

*Thank you for participating in our survey!*
